# Late Reactivation of SARS-CoV-2: A Case Report

**DOI:** 10.3389/fmed.2020.00531

**Published:** 2020-08-20

**Authors:** Antonietta Coppola, Anna Annunziata, Novella Carannante, Valentina Di Spirito, Giuseppe Fiorentino

**Affiliations:** ^1^Department of Respiratory Pathophysiology, Monaldi - Cotugno Hospital, Naples, Italy; ^2^First Division Infectious Disease, Cotugno Hospital, Naples, Italy

**Keywords:** COVID 19, reactivation, naso pharingheal swab, IGM SARS-CoV2, IGG SARS-CoV2

## Abstract

SARS-CoV-2 is a betacoronavirus that belongs to the family Coronaviridae and the order Nidovirales (1). During December 2019, a series of pneumonia cases caused by a SARS-CoV-2 outbreak was identified in Wuhan, Hubei, China, and rapidly spread across the world. The spectrum of SARS-CoV-2 disease (COVID 19) varies from asymptomatic or paucisymptomatic forms to clinical conditions characterized by respiratory failure that necessitate mechanical ventilation and support in an intensive care unit (ICU), multiorgan and systemic manifestations, and, in terms of sepsis, septic shock, and multiple organ dysfunction syndromes (MODS) (2). Whilst many reports have characterized the clinical, epidemiological, laboratory, and radiological features, as well as treatment and clinical outcomes of patients with COVID-19 pneumonia, information on SARS-CoV-2 reactivation remains unreported. Curative and eradicative therapy for COVID-19 is not currently available (3). We report a case of a patient with PCR-confirmed COVID-19 pneumonia who experienced reactivation after 43 days and negative PCR sampling.

## Introduction

We present a case of a male with a history of infection by SARS-CoV-2 (COVID-19) treated with lopinavir/ritonavir, hydroxychloroquine, and tocilizumab. After therapy, the symptoms resolved, and two consecutive nasopharyngeal swabs for SARS-CoV-2 were negative. The patient was considered clinically cured and discharged home. A total of 16 days after discharge and 43 days from the appearance of symptoms, the patient presented with diarrhea, and fever. Presenting with persistent diarrhea and the development of new symptoms, such as asthenia and myalgia, he went to the emergency room where a repeat nasopharyngeal swab for SARS-CoV-2 gave a positive result.

## Case Report

A 68-year-old male (former smoker) presented in the emergency room with diarrhea, asthenia, and fever (37.5°C). The patient's past medical history was remarkable for dyslipidemia, chronic ischemic heart disease, and carbohydrate intolerance.

**Figure 1:**
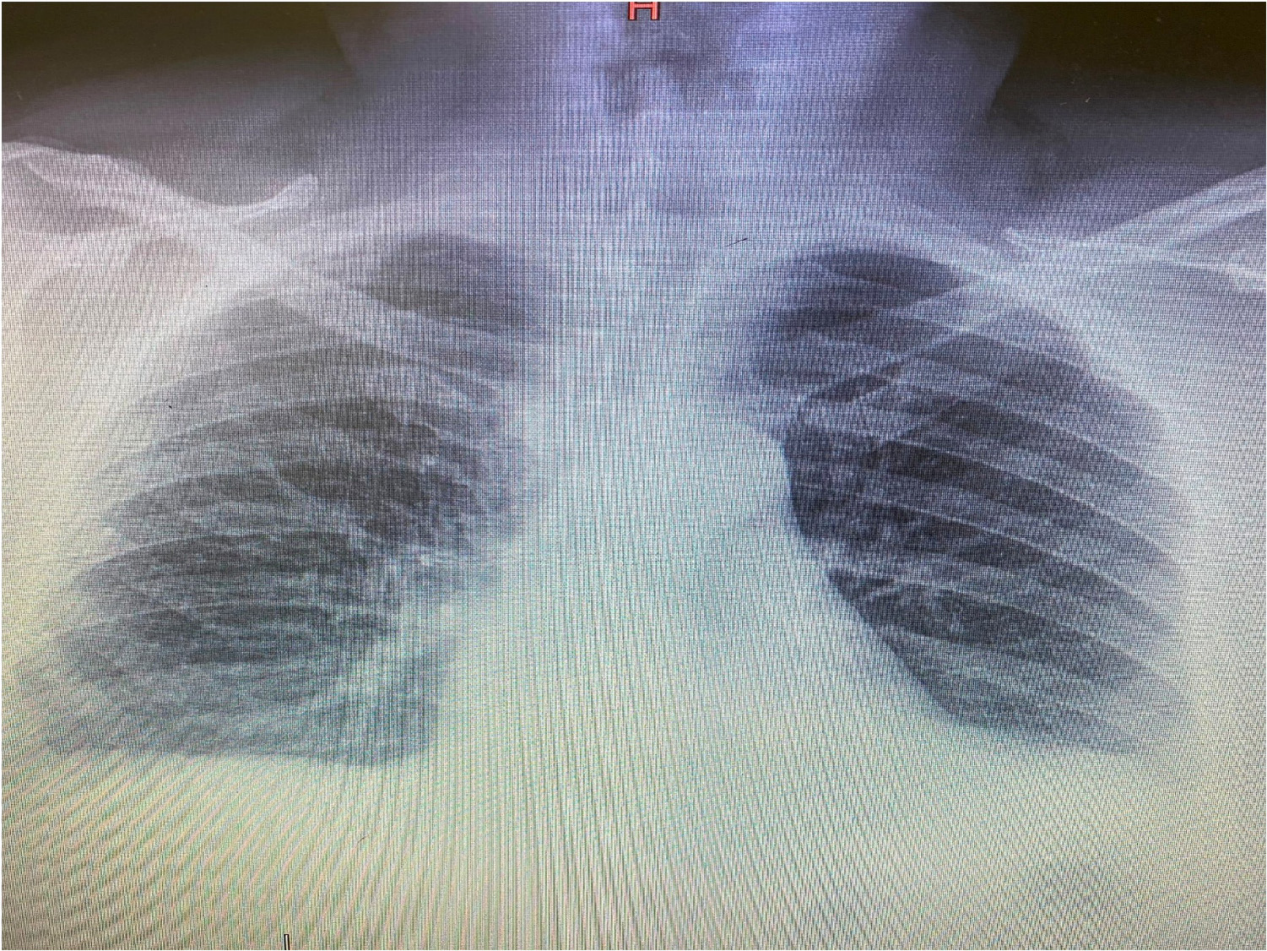
Initial chest X-ray.

The patient's recent clinical history had begun on March 9 with the appearance of a fever (38°C), dry cough, myalgia, and asthenia. On the 12th day of illness, there was a worsening of clinical conditions with the appearance of dyspnea, and so the patient was hospitalized in the infectious diseases department. There, he tested positive for SARS-CoV-2 by PCR from a nasopharyngeal swab, and his chest x-ray revealed bilateral infiltrates as shown in Figure 1. The Interleukine−6 serum level was 41.2 pg/ml. Immunoglobulin M (IgM) for SARS-Cov-2 was 2.51 mg/dl, and Immunoglobulin G (IgG) for SARS-Cov-2 was 21.7 mg/dl.

During hospitalization, he was treated with lopinavir/ritonavir 200/50 mg, two tablets twice daily for 7 days, and hydroxychloroquine 400 mg, one tablet twice daily for 10 days, together with supportive oxygen with nasal cannula at flow 2 (two) liter/min. On the 5th day of hospitalization he received a dose of tocilizumab (8 mg/kg).

**Figure 2:**
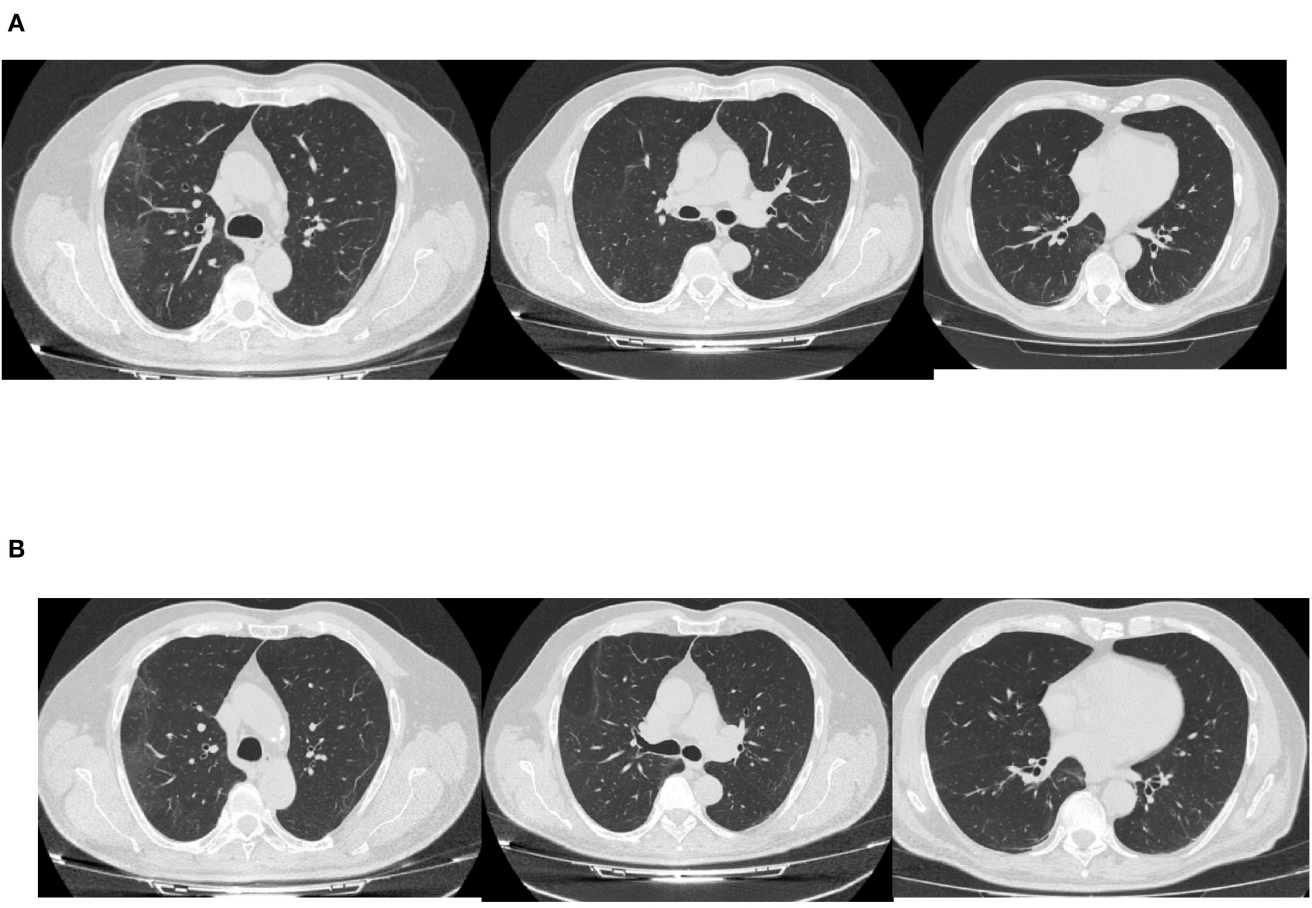
CT scan at day 27 **(A)** and 49 of illness **(B)**.

By the 27th day of illness, the fever and respiratory symptoms had resolved. A CT scan revealed a bilateral interstitial-alveolar pneumonia of a viral nature in the resorption phase (total severity score sec. Chung: 4/20) as shown in Figure 2. C-reactive protein (CRP) was negative, IL-6 levels was 4.7 pg/ml, IgM for SARS-CoV2 were 0.73 mg/dl, IgG for SARS-CoV2 were 49.5, and nasopharyngeal swabs for SARS-CoV-2 were taken serially on the April 2 and 3, resulting in negative PCRs. The patient was considered cured and discharged home on April 4.

On the 16th day after discharge, the patient developed diarrhea. According to the family physician, the patient had a negative result for PCR SARS-CoV-2 via a nasopharyngeal swab. Due to persistent diarrhea and new symptoms of fever, asthenia, and myalgia, the patient went to the emergency room and was admitted to our division (49 days after the first appearance of COVID symptoms). The patient was febrile (37.8°C) and had a blood pressure of 125/75 mmHg, a heart rate of 95 bpm, and a respiratory rate of 20 bpm.

At admission, the laboratory results showed a raised CRP (5.4 mg/dL), creatinine level of 0.7 mg/dL, a sodium level of 143 mmol/L, a glycemia of 127 mg/dl, lymphocyte count of 2.59 × 10^3^ u/L, IL-6 of 15.8 pg/ml, IgM for SARS-CoV-2 of 1.51 mg/dl, and IgG of 25.9 mg/dl.

A nasopharyngeal swab resulted in a positive PCR result for SARS-CoV-2. Arterial blood gas analysis on ambient air revealed normal values: pH 7.41, PO_2_ 84 mmHg, PCO_2_ 34 mmHg, lactate 1.5, and P/F 400. A CT scan documented a reduction in ground glass area as shown in Figure 2.

After discussion in the multidisciplinary team, further escalation of treatment was omitted, except for prophylaxis treatment for deep vein thrombosis.

After 6 days, the fever and other symptoms had resolved. Nasopharyngeal swabs for SARS-CoV-2 were taken serially on May 1 and 2, resulting in negative PCRs; CRP values were normal and the patient was discharged home. The patient lived with his wife who was subjected to two nasopharyngeal swabs for SARS- CoV-2 after 1 week that proved negative the timeline disease is shown in Table 1.

**Table 1:** Case timeline.

**Illness day**	**1^**°**^**	**12^**°**^**	**27^**°**^**	**28^**°**^**	**43^**°**^**	**49^**°**^**	**55^**°**^**
Symptom	Fever Dry cough Myalgia Asthenia	Fever Dry cough Myalgia Asthenia Dyspnoea	No	No	Diarrohea	Diarrohea Fever Myalgia Asthenia	No
C-RP Levels	N/A	Elevated	Normal	Normal	N/A	Elevated	Normal
SARS- CoV 2 PCR on nasopharingal swab	N/A	Positive	Negative	Negative	Negative	Positive	Negative
CT scan	N/A	N/A	Bilateral interstizial pneumonia with ground glass area in resorption phase	N/A	N/A	Reduction in ground glass area	N/A
IL-6 levels	N/A	41.2 pg/ml	4.7 pg/ml	N/A	N/A	15.8 pg/ml	N/A
IGM SARS-CoV2 levels	N/A	2.51 mg/dl	0.73 mg/dl	N/A	N/A	1.51 mg/dl	N/A
IGG SARS-CoV2 levels	N/A	21.7 mg/dl	49.5 mg/dl	N/A	N/A	25.9 mg/dl	N/A

*N/A, not available*.

## Discussion

The literature is rich in the study of the epidemiological, clinical, and radiological features of patients affected by SARS- CoV-2 pneumonia, but there are few reports about SARS-CoV-2 reactivation.

Ye et al. (3) analyzed a group of 55 patients affected by SARS-CoV-2 pneumonia, and they observed that 9% (5 pts) who were discharged from hospital presented with SARS-CoV-2 reactivation. However, they noted that there might be no specific clinical characteristics to distinguish these patients (3), and there is a lack of data about how many days later the symptoms develop again.

Ravioli et al. (4) described two case reports on reactivation of COVID-19 pneumonia, and they concluded that caution should be exercised especially in vulnerable patient populations even after they appear to have overcome the infection; close monitoring on an outpatient basis is therefore crucial (4).

Recently, several studies characterizing adaptive immunoresponses to SARS-CoV-2 infection have reported that most COVID-19 convalescent individuals have detectable neutralizing antibodies that correlate with the numbers of virus-specific T cells. In a recent study, Long et al. observed that IgG levels and neutralizing antibodies in a high proportion of individuals who recovered from SARS-CoV-2 infection start to decrease within 2–3 months after infection (5). This suggest that there is a short duration of immunity after SARS-CoV-2 infection, but we needed additional studies to determinate the duration of antibodies-mediated immunity.

Our case report is very suggestive, because 27 days after the onset of symptoms and treatment with lopinavir/ritonavir 200/50 mg two tablets twice daily for 7 days, hydroxychloroquine 400 mg one tablet twice daily, and a dose of tocilizumab 8 mg/kg on the 5th day, we observed symptom resolution, normal values of CRP, and two negative consequential nasopharyngeal swabs for SARS-CoV-2. The patient was cured. A total of 16 days after discharge, and 43 days from the onset of disease, symptoms appeared again, even if they were milder, and these were associated with an increase in C-RP, IL-6, and IgM for SARS-CoV2 levels. A reactivation of SARS-CoV-2 must be considered and is a very singular reactivation 43 days after the initial onset of symptoms. After discussion in the multidisciplinary team, further escalation of treatment was omitted; an exception was made for prophylaxis treatment for deep vein thrombosis due to mild symptoms and partial radiologic resolution of pneumonia.

Usually, subjects that can be predisposed to reactivation are those with compromised immune systems such as diabetics, dialysis patients for severe renal failure, patients with chronic lung diseases, and cancer patients undergoing immunosuppressive or immunodepressive therapy.

This observation suggests that recovered patients may still be carriers of the virus and require an additional round of viral detection and isolation. Therefore, close monitoring of discharged patients is crucial. Re-infection can be prevented by vaccination, but not completely. Data on this are not yet available, and, furthermore, to understand if a vaccine is really effective it must be tried in the field.

## Data Availability Statement

The raw data supporting the conclusions of this article will be made available by the authors, without undue reservation.

## Ethics Statement

Written informed consent was obtained from the individual(s) for the publication of any potentially identifiable images or data included in this article.

## Author Contributions

All authors listed have made a substantial, direct and intellectual contribution to the work, and approved it for publication.

## Conflict of Interest

The authors declare that the research was conducted in the absence of any commercial or financial relationships that could be construed as a potential conflict of interest.
